# Hepatitis C Virus and Molecular Mimicry

**DOI:** 10.3390/pathogens13070527

**Published:** 2024-06-22

**Authors:** Lynette Goh, Nanda Kerkar

**Affiliations:** 1 KK Women’s and Children’s Hospital, Singapore 229899, Singapore; 2 Massachusetts General Hospital for Children, Harvard Medical School, Boston, MA 02114, USA; nkerkar@mgh.harvard.edu

**Keywords:** hepatitis C, molecular mimicry, autoimmunity

## Abstract

This review delves into the interactions between hepatitis C virus (HCV) and the host immune system, shedding light on how by using the mechanism of molecular mimicry, the virus strategically evades the immune system, resulting in a cascade of diverse complications. HCV, notorious for its ability to persistently infect hepatocytes, employs molecular mimicry to resemble host proteins, thereby avoiding immune detection and mounting an effective defense. This mimicry also triggers systemic autoimmune responses that lead to various sequelae. The objective of this review is to comprehensively explore the role of HCV-induced molecular mimicry, which not only facilitates viral survival but is also instrumental in developing autoimmune and inflammatory disorders. By mimicking host proteins, HCV triggers an immune response that inadvertently attacks the host, fostering the development of autoimmune and other inflammatory disorders. Understanding the nuanced mechanisms of HCV-mediated molecular mimicry provides crucial insights into the multifaceted sequelae of viral infections on host immune responses. Unravelling these complexities is paramount for advancing therapeutic strategies that not only target the virus directly but also mitigate the secondary autoimmune and inflammatory complications induced by HCV.

## 1. Introduction

The human body’s immune system is responsible for differentiating between self and non-self as part of its defense mechanism against external antigens. The induction of anergy occurs when tolerance is established to self-antigens, either through inaccessibility or low levels of antigen [1]. There are shared amino acid sequences between foreign and self-antigens, leading to cross-reactivity when pathogen-specific immune responses also attack host structures and result in the development of autoimmunity. Molecular homology between virus and host challenges the homeostatic mechanisms that allow for immune tolerance. Molecular mimicry can thus act as a direct mechanism for inducing autoimmunity, in addition to the indirect stimulation of autoreactive lymphocytes.

In molecular mimicry, the immune response against a foreign virus cross-reacts with host tissue antigens, causing tissue destruction. This autoimmune attack may progressively spread to other self-antigens. This is due to the ability of the immune response to propagate to alternative sites on an antigen, otherwise known as epitope spreading [2]. While epitope spreading is an efficacious mechanism to clear foreign antigens, it may also result in the intensification of an autoimmune reaction [3]. Furthermore, some activating peptides are not completely identical to host sequences, which makes the prediction and detection of critical epitopes challenging.

Additionally, in the process of viral infection, formerly quiescent autoreactive T- and B-cells may become activated with the upregulation of the related costimulatory processes, setting off a cascade of inflammation. Self-antigens that were previously not accessible to the immune system may become immunologically active upon the initiation of inflammation, resulting in the expansion of a proinflammatory state and its related cytokines, which in turn leads to an imbalance of immunoreactivity and anergy.

This review aims to describe how the mechanism of molecular mimicry used by the hepatitis C virus (HCV) to evade the host immune system inadvertently causes a range of organ- and nonorgan-specific autoimmune disorders.

## 2. HCV and Immune Evasion

Molecular mimicry, where viral proteins that are structurally similar to host defense proteins are expressed, confers an important immune escape strategy. HCV is well known to resist treatment with interferon (IFN) by using this mechanism [4].

IFN acts in part through the dsRNA-dependent protein kinase (PKR) to inhibit protein synthesis through the phosphorylation of eukaryotic initiation factor 2α (eIF2α). The HCV envelope protein E2 contains a 12-aa sequence identical to the phosphorylation domains of both eIF2α and the PKR kinase [5]. This domain prevents the PKR-dependent phosphorylation of eIF2α and the inhibition of protein synthesis. The extent of the PKR-eIF2α homology of this domain correlates with the ability of HCV to resist type I IFN treatment. This ability was reproducible in transgenic mammalian cells with HCV nonstructural protein 5A (NS5A), showing a repression of PKR [6] as well as NS5A expressing human cell lines where NS5A expression can confer IFN resistance [7].

In addition, the N-terminal region E2 of HCV has high levels of homology with human immunoglobulin (Ig) variable domains. E2 also has common amino acid (aa) sequence features not only of regions of human Ig light chains, but also heavy chains and T cell receptors. Using a position specific scoring system, the degree of homology correlates with immune escape and persistence in humans [8].

Through these strategies employed by the HCV virus, IFN monotherapy, used in the past, has become ineffective in the clearance of HCV. Currently, several direct-acting antiviral agents are available to treat hepatitis C.

## 3. HCV and Autoimmune Disease

**Figure 1:**
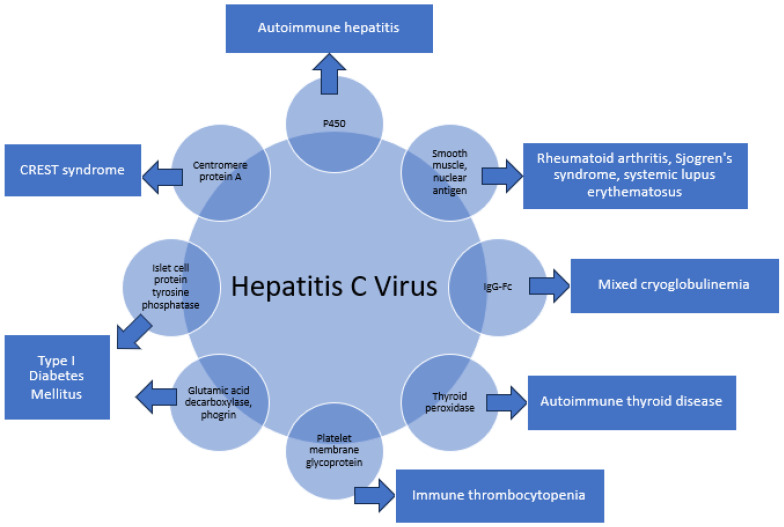
The myriad of structural similarities between HCV and human self-antigens.

HCV has been associated with a spectrum of autoimmune complications. Studies have shown that 40–74% of patients with HCV [9] could develop autoimmune diseases such as autoimmune hepatitis, rheumatoid arthritis, Sjogren’s syndrome, mixed cryoglobulinemia, thyroid disease, and diabetes mellitus. It is postulated that the immunogenic homologous sequences between HCV and oneself, together with the make-up of the host microenvironment, lead to the eventual development of autoimmune disease through molecular mimicry. Figure 1 shows the myriad of structural similarities between HCV and human self-antigens.

### 3.1. Specific Examples of Molecular Mimicry

#### 3.1.1. P450-CYP2D6, CYP2A6, and CYP2A7 8–17 in Autoimmune Hepatitis

Cytochrome P450 (CYP2D6) is the target of liver kidney microsomal autoantibody type 1 (LKM1) in autoimmune hepatitis (AIH) type 2. It has been found that CYP2D6_193–212_ contains the hexameric sequence “RLLDLA” that shares 5 of 6 aas with the “RLLDLS” of HCV_2985–2990_ [10]. Cross-reactive antibody recognition was also demonstrated between HCV core _2985–2990_ and CYP2D6_204–209_ in LKM+ patients with HCV [11]. Moreover, HCV_310–324_ also shares amino acid sequences with the immunodominant region of CYP2D6_254–271_ [12], with simultaneous antibody reactivity demonstrated to both CYP2D6_254–271_ and HCV _310–324_ in LKM1+ HCV-infected patients [13]. It was also found that HCV core _178–187_ shares sequence homology with both CYP2A6 and CYP2A7_8–17_. Subsequently, cytotoxic T lymphocytes (CTLs) that were induced by HCV recognized both CYP2A6 and CYP2A7 peptides as well as the endogenously processed CYP2A6 protein [14]. In both the liver and blood of patients with AIH type 2, CD4+ and CD8+ autoreactive T cells that target CYP2D6 have been detected, indicating a role of cytotoxic T cells in the pathogenesis of this disease.

The CYP2E1_324–346_ peptide also showed homology with HCV_438–449_ and _456–465_ in mouse studies, confirming the cross-reactivity of anti-CYP2E1 IgG with both HCV_438–449_ and _456–465_ [15]. These specific, similar sequences between HCV and P450 have been shown to cause the production of cross-reactive antibodies that may initiate the autoimmune response, manifest as LKM1 antibodies, and possibly progress to autoimmune hepatitis. A child started producing the LKM1 antibody two weeks after being infected by HCV following a liver transplantation for end-stage liver disease secondary to an alpha-1 antitrypsin deficiency. This demonstration of a temporal relationship between HCV infection and the development of LKM lends support to the concept that the two are causally related [16]. Similarly, the development of AIH type 2 was reported in a nurse with a predisposing HLA haplotype who had a needle-stick injury while caring for a patient infected with HCV [17]. The clinical implications of developing autoantibodies can be seen in LKM-positive HCV patients who showed more severe histological findings and higher liver fibrosis scores than those who were LKM-negative [18,19]. It is important to understand the contribution to liver damage caused by HCV-induced autoimmunity.

#### 3.1.2. Human Nuclear and Smooth Muscle Antigens in Rheumatoid Arthritis, Sjogren’s Syndrome, and Systemic Lupus Erythematosus (SLE)

It has been shown that the odds of HCV in patients with SLE and Sjogren’s syndrome are almost three times that of the general population [20]. Antinuclear antibodies (ANAs) and smooth muscle antibodies (SMAs) are commonly seen in patients with HCV. Amongst patients with HCV, 10–30% develop ANA positivity [21,22]. There are three smooth muscle (smoothelin698–717, myosin1035–1054, vimentin69–88) and three nuclear (matrin722–741, histone H2A11–30, replication protein A133–152) antigens identified that have close homology with HCV, where cross-reactive immune responses have been demonstrated [23]. This gives strength to the hypothesis that in HCV, the mechanism of molecular mimicry might predispose to the development of autoimmune diseases such as rheumatoid arthritis and Sjogren’s syndrome, characterized by ANA and/or SMA positivity.

#### 3.1.3. IgG-Fc in Mixed Cryoglobulinemia

Cryoglobulins are abnormal proteins that reversibly precipitate at reduced temperatures. Cryoglobulinemia can be classified into three types, namely type I, II, and III. Types II and III comprise mixed cryoglobulinemia (MC) and are most frequently related to HCV, with HCV infection often thought to be an inducing trigger. Epidemiological studies have shown that 92% of patients with MC also have chronic HCV [24]. Circulating mixed cryoglobulins, composed of immune complexes of polyclonal IgG and monoclonal IgM with rheumatoid factor (RF) activity, are detected in 40–60% of patients with chronic HCV infection [24].

Mixed cryoglobulins usually contain IgM and IgG immunoglobulins, with IgM having rheumatoid factor activity directed against IgG molecules. The underlying pathophysiology behind this may be due to the presence of shared epitopes between the HCV and IgG-Fc domains that result in cross-reactivity [25], with evidence for this having been demonstrated in several MC patients between HCV-NS3 and IgG-Fc, specifically HCV NS (31250–1334) and IgG-Fc (345–355) [26]. This leads to immune complex formation, cryoprecipitation, and consequently glomerulonephritis or vasculitis. There are also structural similarities between the HCV core antigen and complement protein C1q, which would explain the presence of cross-reactive anti-C1q in HCV-associated mixed cryoglobulinemia [27]. Molecular mimicry may complement epigenetic susceptibilities as causative mechanisms for mixed cryoglobulinemia.

The immediate priority in the treatment of HCV-associated mixed cryoglobulinemia is the eradication of HCV with direct-acting antivirals. This has been shown to be very effective, with either a partial (20%) or complete disappearance of vasculitis symptoms (65%). However, despite sustained virological response, cryoglobulinemia vasculitis may persist or reappear over variable lengths of time from the completion of therapy [28].

#### 3.1.4. Thyroid Peroxidase (TPO) Peptides in Hashimoto’s and Grave’s Disease

The thyroid is one of the principal organs involved in extrahepatic manifestations of chronic HCV. In pediatric studies, 20% of children with HCV had anti-TPO antibodies [29]. Bogdanos et al. demonstrated cross-reactive antibody responses to HCV and TPO peptides in patients with HCV infection [30]. Subsequent treatment of HCV with IFN potentiates autoimmune thyroid disease by stimulating host immune responses. IFN activates the JAK–STAT pathway, leading to the further production of cytokines, chemokines, and adhesion molecules. The effect of IFN treatment can be predicted by pretreatment anti-TPO positivity [31]. The underlying causality of autoimmune thyroid disease in patients with HCV could be attributed to the structural similarities between HCV and TPO peptides. HCV patients on IFN should have their thyroid status regularly screened and undergo appropriate treatment to maintain a clinically euthyroid state.

#### 3.1.5. Platelet Membrane Glycoprotein IIIa (GPIIIa49–66) in Immune Thrombocytopenia

HCV contains proteins with peptide sequences homologous to platelet membrane glycoprotein IIIa GPIIIa49–66 [32]. It is postulated that the presentation of these peptides to the host immune system results in the production of antibodies directed against GPIIIa49–66 by some individuals. The antibodies then react with GPIIIa on autologous platelets, leading to platelet fragmentation and thrombocytopenia. The possibility of molecular mimicry suggests the need for an investigation into peptide mimics for immunogenicity and pathogenicity in further experimental and human models.

#### 3.1.6. Glutamic Acid Decarboxylase 65-Kilodalton Isoform (GAD65), Protein Tyrosine Phosphatase Islet Cell Antigen-2, and Phogrin in Type I Diabetes Mellitus (DM)

There have been reports of type I DM associated with HCV. There was a male patient who developed acute hepatitis C after a blood transfusion. Four weeks post-transfusion, he developed GAD antibodies and islet cell antibodies, and then developed type I DM a year later [33]. There are a significant number of case reports of type I DM in patients with HCV after treatment with IFN [34]. These patients present with overt signs of diabetic ketoacidosis with polyuria, polydipsia, hyperglycemia, and severe metabolic acidosis. The levels of GAD antibodies were reported to be significantly higher in IFN-induced type I DM compared to classic type I DM [35]. For those who were tested for antibodies, markers for pancreatic islet autoimmunity were present prior to IFN therapy in 50% of cases with IFN-induced type I DM [36].

The immunostimulatory effect of IFN likely played a part through inducing and accelerating an underlying diabetogenic process in HCV. Unfortunately, the cessation of IFN does not reverse the process, and patients would still be insulin-dependent [37]. Although IFN is no longer the first line treatment for HCV, it is recommended that patients with HCV who are considering treatment with IFN undergo screening for GAD and islet cell autoantibodies prior to treatment initiation [38]. Interestingly, there are amino acid similarities between HCV and GAD65 and protein tyrosine phosphatase islet cell antigen-2 and phogrin [39]. Molecular mimicry could be a mechanism that complements immune transformations while developing an autoimmune response in these cases.

#### 3.1.7. Centromere Protein-A (CENP-A) in CREST Syndrome

CREST syndrome, an acronym for calcinosis, Raynaud’s phenomenon, esophageal dysmotility, sclerodactyly, and telangiectasia, is characterized by the presence of the anticentromere antibody (ACA). Molecular similarities between the HCV core antigen and CENP-A [40], one of the major centromere proteins [41], have been shown, with anticentromere antibodies being positive in 1% of patients with HCV [42].

## 4. Direct-Acting Antiviral Agents

Direct-acting antiviral agents are now the first-line therapy for HCV, as they inhibit viral replication, unlike IFN, which inhibits protein synthesis and stimulates host immune responses [43]. Unfortunately, it is still possible to develop autoimmune disease after the treatment of HCV with direct-acting antiviral agents with a highly sustained virological response. Morihisa et al. described autoimmune hepatitis and primary sclerosing cholangitis despite the clearance of HCV with direct-acting antiviral agents and undetectable serum HCV RNA [44]. This may suggest the persistence of autoantibodies despite the elimination of HCV.

## 5. Other Viruses with Molecular Homology and Associated Autoimmune Conditions

**Figure 2:**
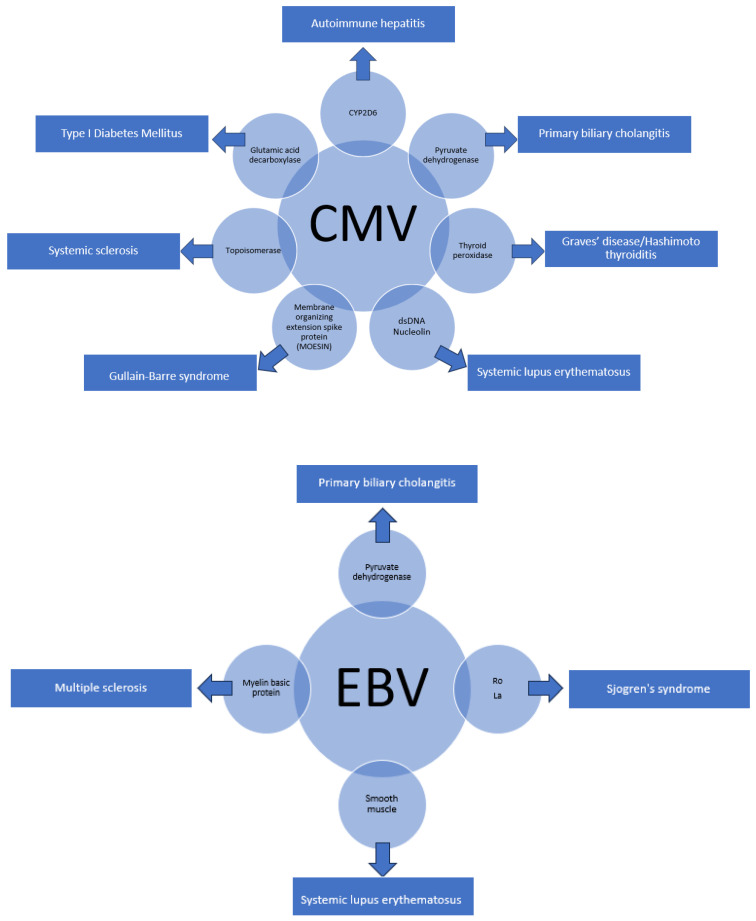
Other Viruses with Molecular Homology and Associated Autoimmune Conditions.

Cytomegalovirus (CMV) and Epstein–Barr virus (EBV) have also been implicated in the development of autoimmune diseases due to the presence of molecular similarities between the viruses and human self-antigens. Figure 2 below shows the overlaps that have been identified thus far.

## 6. Conclusions

The complete characterization of the entire HCV polyprotein in 1993 [45] was a breakthrough that helped build the foundation of our understanding of the disease. However, linear peptide homology is not the only mechanism by which molecular mimicry can cause unforeseen complications. Peptide modelling with three-dimensional structural analysis, molecular docking analysis, and affinity estimation is a potential strategy through which molecular mimicry may cause unintended diseases. Hence, the current approaches to studying autoimmunity associated with molecular mimicry need to be further expanded to include these to be able to identify the complex pathophysiology underlying this phenomenon.

As evidence of causality, in addition to antibodies or cross-reactive T cells, HCV has structurally similar epitopes. Furthermore, temporal links where preceding exposure to HCV leads to the subsequent development of autoimmune disease suggest a causal role in individuals who may be predisposed to the development of autoimmunity. New viruses have been identified in the potentiating role of viral infections, leading to autoimmunity. New onset autoantibodies have been found in acute coronavirus disease 2019 (COVID-19) infections [46], and case reports are emerging regarding the appearance of autoimmune diseases post-COVID-19 infection [47,48]. New diagnoses of autoimmune diseases as well as flares of pre-existing autoimmune conditions have also been described after the COVID-19 vaccination [49]. This warrants close attention and further investigations to better understand the immunogenicity and pathogenicity of the implicated homologues, along with the factors that affect their potency as triggering peptides.
